# Partial Gallbladder Avulsion Following Blunt Abdominal Trauma: A Case Report

**DOI:** 10.7759/cureus.94609

**Published:** 2025-10-15

**Authors:** Cláudia Lima, Telma Brito, Fábio Viveiros, José Couto, Teresa Almeida

**Affiliations:** 1 General Surgery, Unidade Local de Saúde do Alto Minho (ULSAM), Viana do Castelo, PRT

**Keywords:** biliary trauma, blunt abdominal trauma, gallbladder avulsion, gallbladder injury, hepatic laceration, laparoscopic cholecystectomy, motorcycle accident

## Abstract

Gallbladder injuries secondary to blunt abdominal trauma are uncommon and often associated with other intra-abdominal lesions. Due to nonspecific clinical findings and subtle radiologic features, diagnosis is frequently delayed, which may contribute to increased morbidity if surgical treatment is not promptly instituted.

We report the case of a young male who sustained blunt abdominal trauma following a motorcycle accident and presented with persistent abdominal pain. Imaging raised suspicion of acute cholecystitis, but diagnostic laparoscopy revealed a partial gallbladder avulsion associated with a hepatic laceration and active bleeding. Laparoscopic cholecystectomy was performed successfully, with hemostasis achieved and no need for conversion to laparotomy. The postoperative course was marked by transient hematobiliary drainage but was ultimately uneventful, and the patient was discharged in good condition. Histopathology confirmed a congested and hemorrhagic gallbladder wall without calculi.

This case highlights the diagnostic challenges of gallbladder trauma in the absence of specific clinical or imaging findings and emphasizes the importance of considering this rare injury in patients with blunt abdominal trauma. Early surgical intervention in stable patients may provide definitive management with favorable outcomes.

## Introduction

The most frequently involved organs in blunt abdominal trauma are the liver, spleen, and kidneys [1]. By contrast, the gallbladder is relatively well protected due to its anatomical position, being partially embedded within the liver parenchyma and shielded by the omentum, intestinal loops, and rib cage [2]. For this reason, gallbladder injuries are rare and are usually associated with other concomitant intra-abdominal injuries [2]. The reported incidence of blunt gallbladder trauma ranges between 0.5% and 2.1% [1]. Diagnosis can be challenging and is often delayed, which may result in progression to peritonitis with nonspecific clinical manifestations such as nausea, vomiting, weight loss, jaundice, ascites, fever, abdominal pain, or distension [3]. These injuries occur more frequently in males, with a median reported age of 27 years [4]. Contusions and intramural hematomas may occur and are often under-reported, as they are usually diagnosed intraoperatively [5]. Children are also at increased risk because of their susceptibility to direct abdominal trauma and the relative underdevelopment of their anterior abdominal wall musculature [6].

## Case presentation

A 21-year-old man presented to the emergency department 48 hours after a low-speed motorcycle accident with complaints of severe abdominal pain localized to the umbilical region. On examination, he was tachypneic but hemodynamically stable, with diffuse abdominal tenderness more pronounced in the epigastrium and right upper quadrant. Laboratory tests showed hemoglobin 16.3 g/dL, leukocytes 15,640/µL, C-reactive protein 2.85 mg/dL, total bilirubin 1.9 mg/dL, AST 181 U/L, and ALT 290 U/L (Table 1). Serum proteins (albumin and total protein) were not obtained at admission.

**Table 1:** Laboratory findings on admission AST: Aspartate aminotransferase; ALT: Alanine transaminase.

Test	Patient Value	Reference Range
Hemoglobin	16.3 g/dL	13.0–17.0 g/dL
Leukocytes	15,640/µL	4,000–11,000/µL
C-reactive protein	2.85 mg/dL	<0.5 mg/dL
Total bilirubin	1.9 mg/dL	0.2–1.2 mg/dL
AST	181 U/L	10–40 U/L
ALT	290 U/L	10–55 U/L

**Figure 1:**
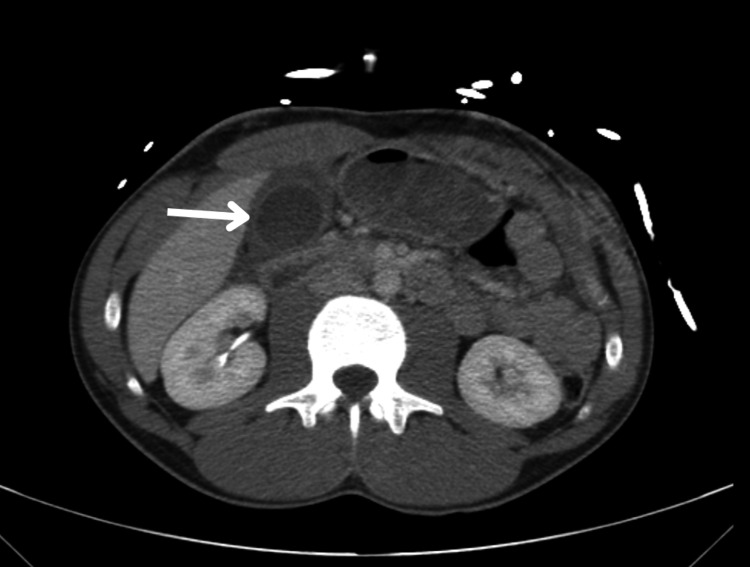
Axial computed tomography image demonstrating the distended gallbladder without calculi with circumferential wall thickening (arrow)

**Figure 2:**
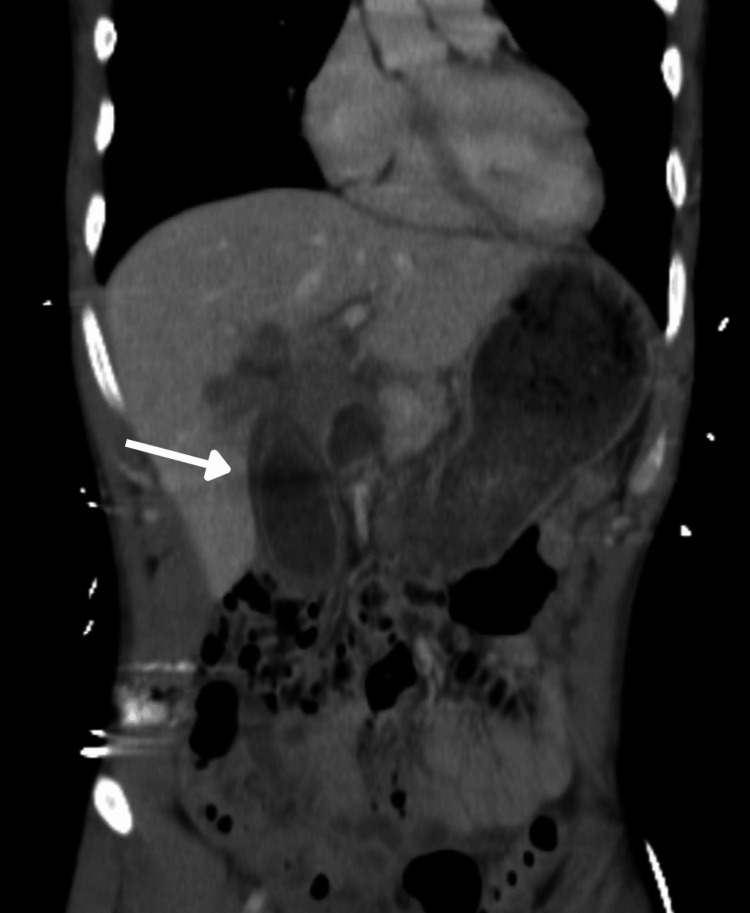
Coronal computed tomography showing wall thickening and mild pericholecystic stranding (arrow)

Computed tomography (CT) revealed a bilobed and distended gallbladder without stones, with a thickened wall, reported as possible acute cholecystitis, and no other thoraco-abdominopelvic abnormalities (Figures 1, 2). On consensus re-review with radiology, a superficial capsular laceration of segment IVa-IVb contiguous with the gallbladder bed was suspected, and there was no active contrast extravasation or pseudoaneurysm on the admission CT. Edematous thickening was localized to the gallbladder wall (predominantly subserosal) with mild pericholecystic stranding, without generalized visceral edema. No other intra-abdominal injuries were identified on the initial assessment.

**Figure 3:**
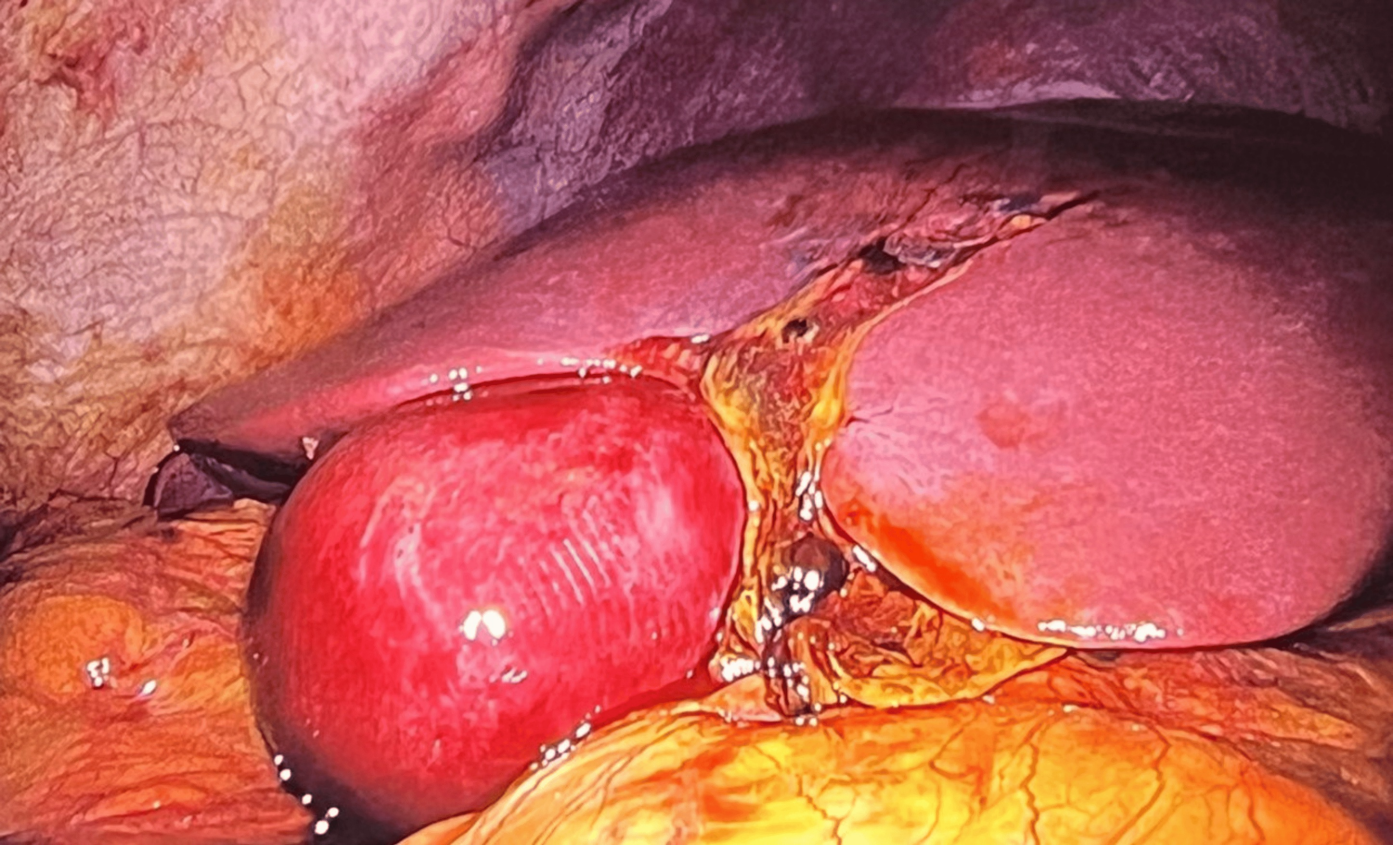
Intraoperative laparoscopic view showing liver laceration and gallbladder with hemorrhagic appearance

**Figure 4:**
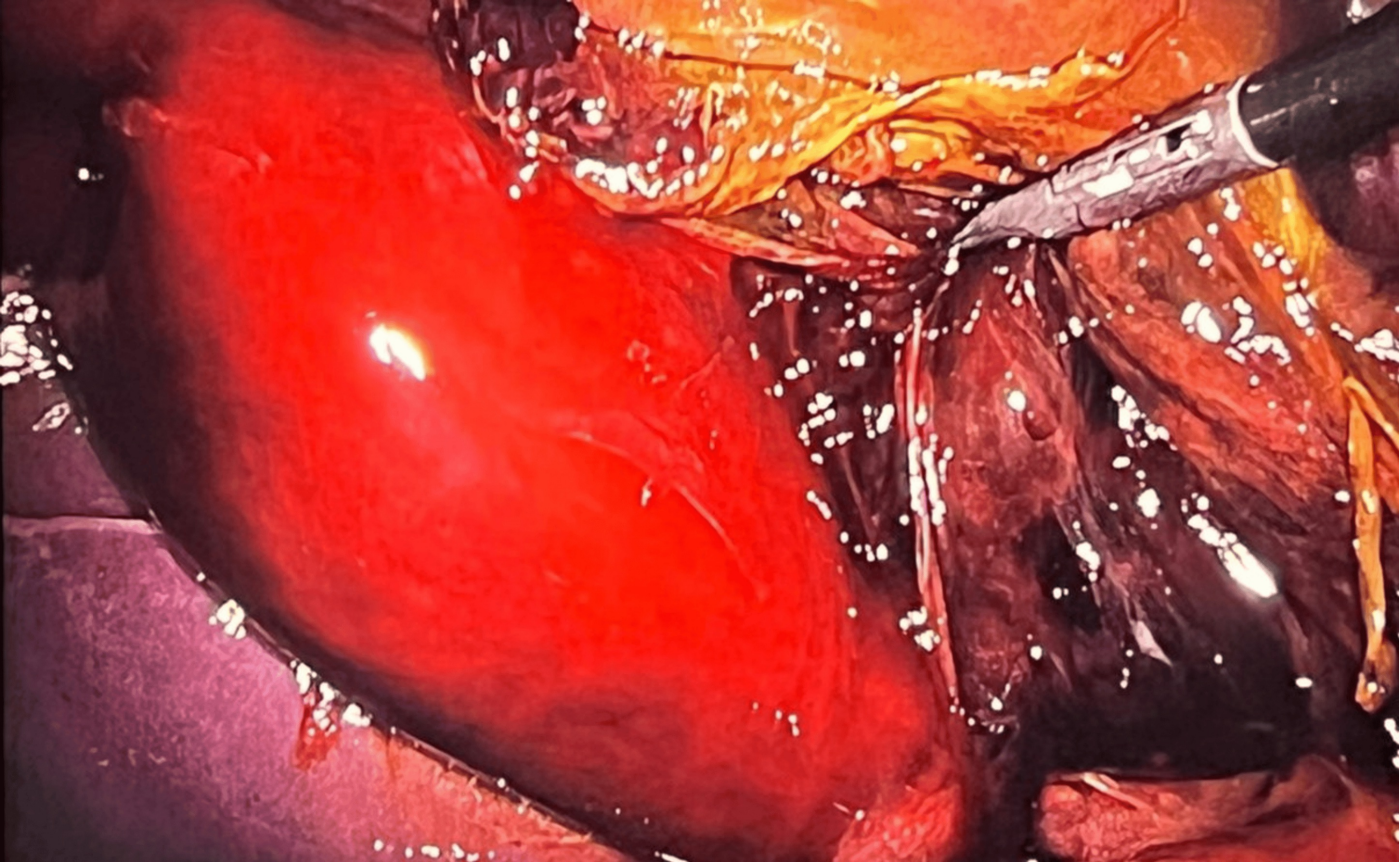
Intraoperative laparoscopic image revealing gallbladder with partial avulsion of the hepatic bed

Due to persistent abdominal pain, surgical intervention was proposed. Given the patient’s hemodynamic stability, a laparoscopic approach was chosen. Intraoperatively, a small-volume hemoperitoneum was observed, as well as partial avulsion of the gallbladder, accompanied by a segment IV liver laceration with active bleeding and bile leakage (Figures 3, 4). Bleeding was consistent with low-pressure parenchymal oozing from the segment IV laceration contiguous with the gallbladder bed; no discrete arterial bleeder was identified.

**Figure 5:**
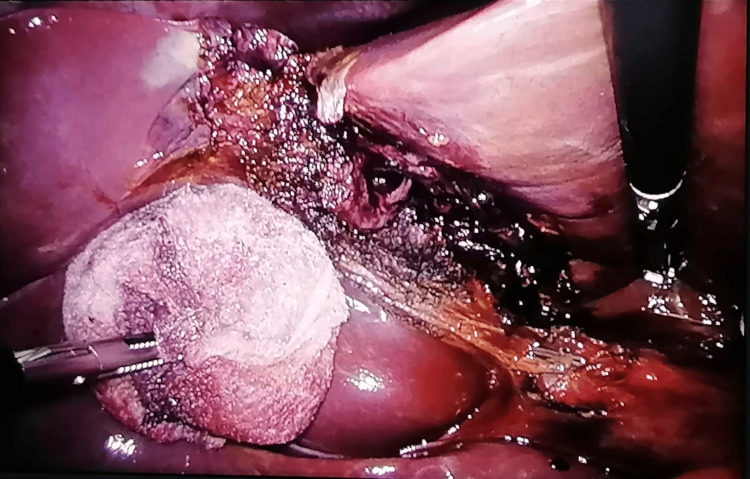
Laparoscopic view of liver bed after cholecystectomy/partial avulsion, demonstrating the avulsion plane at segment IV with adherent clot; no active arterial bleeding was observed.

Laparoscopic cholecystectomy was performed with adequate hemostasis, without the need for conversion to open surgery (Figure 5). A drain was placed in the gallbladder bed.

On the second postoperative day, the drain output was approximately 650 mL of hematobiliary fluid, which progressively decreased. The patient remained hemodynamically stable throughout. On the fourth postoperative day, worsening abdominal pain prompted a repeat CT, which demonstrated a grade III/IV liver laceration involving the hilar region and the posterior aspects of segments I and VIII. At the surgical bed, a sentinel clot was identified with a slight increase in the adjacent liver laceration, but no active hemorrhage was seen. Conservative management was adopted. The remainder of the hospitalization was uneventful, and the patient was discharged on the 11th postoperative day in stable condition.

Histopathologic examination of the surgical specimen revealed a gallbladder measuring 8 cm in length, with smooth serosa interspersed with hemorrhagic areas. The wall was thin (maximum thickness 0.3 cm), and the mucosa appeared greenish and granular, with no stones. Microscopy showed congestion, edema, and hemorrhage, particularly in the subserosa, without mucosal alterations. These microscopic findings are compatible with a gallbladder contusion and coexisted with the macroscopic partial avulsion.

## Discussion

Gallbladder injuries resulting from blunt abdominal trauma are classified as contusion, perforation, or avulsion, which may be partial, complete, or total [2]. Contusion corresponds to an intramural hematoma, most often diagnosed intraoperatively and therefore under-reported [5]. Such hematomas may progress to necrosis and subsequent perforation [3]. Perforation, also termed rupture or laceration, is the most frequent injury, usually involving the fundus [4] or the gallbladder neck [7], and is commonly associated with choleperitoneum [8]. Avulsion can be subdivided into partial, in which the gallbladder is partially detached from the hepatic bed; complete, in which the gallbladder is fully detached from the liver bed but remains connected via the cystic duct and artery; and total, also called traumatic cholecystectomy, where the gallbladder is entirely free within the abdominal cavity [2]. Only a few reports in the literature describe these injuries in detail [9,10]. According to the Losanoff and Kjossev classification [11,12], our patient’s injury corresponds to type 3A (Table 2). In practical terms, our case demonstrates a contusion component on histology coexisting with a partial avulsion, a combination that likely reflects a traction/shear mechanism.

**Table 2:** Losanoff and Kjossev classification of traumatic gallbladder injuries Source: [11,12].

Type	Injury of the Gallbladder
1A	Contusion with intramural hematoma
1B	Contusion with perforation
2	Rupture
3A	Avulsion with partial detachment
3B	Avulsion with complete detachment from the liver but attached to the structures of the hepatoduodenal ligament (“near traumatic cholecystectomy”)
3C	Torn only from the hepatoduodenal ligament
3D	Completely torn from all attachments (“traumatic cholecystectomy”)
4A	Traumatic cholecystitis, secondary to hemobilia
4B	Acute acalculous cholecystitis
5	Mucosal tear with leakage of bile

Blunt gallbladder trauma most frequently results from road traffic accidents, followed by falls and direct abdominal blows [2,7]. The gallbladder is particularly vulnerable to avulsion forces due to its thin wall, high distensibility, and lack of strong fixation ligaments. Distension after food intake or increased intraductal pressure caused by alcohol consumption, via contraction of the sphincter of Oddi, may also predispose the gallbladder to rupture [1]. Conversely, a chronically inflamed gallbladder with a thickened wall may have a lower risk of traumatic injury [9].

Concomitant injuries are common, particularly hepatic lacerations, which occur in 83%-91% of cases [2,9]. Duodenal and splenic injuries are also frequently reported [2,9]. In our case, a concomitant hepatic laceration was identified intraoperatively. Diagnosis is often delayed because gallbladder trauma presents with nonspecific symptoms and minimal peritoneal signs, especially when bile leakage is sterile [1]. Laboratory abnormalities may also be absent [9]. As a result, delays of up to six weeks in diagnosis have been described [1].

CT is currently the most reliable imaging modality for gallbladder trauma [9]. However, findings are often nonspecific, including pericholecystic fluid, wall thickening or discontinuity, collapsed lumen, intraluminal clots, arterial extravasation, displacement of the gallbladder, or mass effect on adjacent organs [1,2]. Liver injuries may overshadow gallbladder lesions on CT, which explains why many injuries are only diagnosed during surgery [3]. Ultrasound can show pericholecystic fluid, a thickened wall, heterogeneous intraluminal content, or discontinuity of the wall. Nonvisualization of the gallbladder should raise suspicion for avulsion or rupture, although ultrasound is more useful for nontraumatic pathologies. Magnetic resonance cholangiopancreatography (MRCP) provides better anatomical detail of the biliary tree compared with CT but is rarely used in acute trauma. Peritoneal lavage may also support the diagnosis if bile is identified in the fluid, although the absence of bile does not exclude the injury [9]. Hepatobiliary scintigraphy and MRI may further assist in selected cases [10].

Treatment depends on the type and severity of the gallbladder injury, associated lesions, and the patient’s overall condition [10,11]. Minor injuries such as contusions, small lacerations, or isolated partial avulsions can be managed conservatively, occasionally with percutaneous drainage, although delayed necrosis and perforation have been described [2]. Severe injuries generally require cholecystectomy. In hemodynamically stable patients, a laparoscopic approach is feasible, and diagnostic laparoscopy is recommended when the diagnosis is uncertain. Placement of an abdominal drain after cholecystectomy is indicated when there are associated injuries or in subtotal cholecystectomy due to the risk of bile leakage [6]. In our patient, a drain was placed given the associated liver laceration and bile leakage from the avulsion plane.

Limitations

Limitations include the absence of serum protein measurements and the single-case design; nevertheless, the localized pattern and histologic findings support the proposed mechanism and diagnosis.

## Conclusions

Blunt traumatic gallbladder injury is a rare condition that may be overlooked due to its nonspecific clinical manifestations. Delayed diagnosis increases the risk of morbidity and mortality. Prognosis is generally favorable when the injury is recognized early and no significant associated lesions are present. A high index of suspicion is therefore essential, taking into account the mechanism of trauma and clinical presentation. Early imaging and surgical evaluation are critical to avoid missed or late diagnosis and to ensure optimal outcomes. When CT findings are subtle but clinical concern persists in a hemodynamically stable patient, early diagnostic laparoscopy may provide both diagnosis and definitive management.
